# Sports Facilities, Shopping Centers or Homes: What Locations are Important for Adults’ Physical Activity? A Cross-Sectional Study

**DOI:** 10.3390/ijerph13030287

**Published:** 2016-03-04

**Authors:** Marijke Jansen, Dick Ettema, Frank Pierik, Martin Dijst

**Affiliations:** 1 Department of Human Geography and Spatial Planning, Faculty of Geosciences, Utrecht University, Heidelberglaan 2, Utrecht 3584 CS, The Netherlands; d.f.ettema@uu.nl (D.E.); m.j.dijst@uu.nl (M.D.); 2 Department of Urban Environment and Safety, Nederlandse Organisatie voor Toegepast Natuurwetenschappelijk Onderzoek/Netherlands Organisation for Applied Scientific Research (TNO), P.O. Box 80015, Utrecht 3508 TA, The Netherlands; frank.pierik@tno.nl

**Keywords:** physical activity, global positioning system (GPS), accelerometer, activity locations

## Abstract

Physical activity (PA) is influenced by the built environment. However, little is known about the types of built environment where adults spend their time, and at what levels of PA they engage in those environments. Understanding the effect of the built environment on PA requires insight into PA behavior at different types of locations (e.g., home, work, shopping centers, and sports facilities). Therefore, this study describes where adults aged 45–65 years were active with moderate-to-vigorous intensity (MVPA), and examines associations of socio-demographic factors and neighborhood with MVPA at these locations. Participants’ (*N* = 308) PA was measured for seven days using accelerometers and GPS-devices. Adults spent most minutes of MVPA at home and work. Highest MVPA-ratios of total time spent at a location were achieved in sports facilities and during transport. Neighborhood characteristics and socio-demographic factors such as work status, health status and household structure, had significant effects on MVPA at various locations and on total MVPA. Understanding PA behavior at various locations may provide insights that allow professionals in different domains (e.g., health, landscaping, urban planning) to develop strategies to stimulate PA.

## 1. Introduction

The positive effects of physical activity (PA) on physical and mental health [1,2], health-related quality of life [3], and healthy ageing [4] have been extensively documented. However, worldwide, the percentage of adults who do not meet World Health Organization recommendations for PA is still 31.1% [5]. Insufficient PA is seen as a major public health problem, which puts a high demand on society due to the high costs it generates [1,6].

The built environment (*i.e.,* the spatial organization of residential, work, shopping- and recreational areas, their layout and appearance, and the transportation system connecting them) has been identified as a factor that influences PA levels [7,8]. For example, neighborhood walkability and street connectivity have been found to be associated with active transport, and factors such as safe crossings, pavement, greenness, attractiveness, and proximity to facilities for recreation have been identified as correlates of leisure activity [8]. Most research into the relationship between the built environment and health behaviors such as PA has focused on the residential environment [9] and its effect on total PA or on a specific type of PA, such as walking or cycling [7,10,11]. However, as Sallis *et al.* (2006) stated in their ecological model of active living [12], individuals interact with various environments as they participate in different types of activities at different places throughout the day (active transport, occupational activities, household activities and active recreation). This suggests that researchers should study PA behavior in a broader geographical context than the neighborhood alone. Since specific physical activities are often related to certain elements of the built environment such as parks, infrastructure, and building complexes (e.g., infrastructure can influence transport-related PA), understanding the effects of the built environment on PA ideally requires that levels of PA are assessed for specific locations.

The increasing awareness that the impact of the built environment on PA should be understood and measured for specific locations has recently spawned a stream of studies that used accelerometers and GPS-devices to assess location specific PA. Most of these studies have investigated children’s PA behavior. These studies distinguished indoor and outdoor PA behavior, PA within and outside children’s neighborhoods, and (MV)PA at various locations such as the home, gardens, green spaces, schoolyards, playgrounds, sports facilities and streets (see e.g., [13,14,15,16,17,18,19]). In addition, Dunton *et al.* (2013) investigated the locations (e.g., residential locations, public facilities and open spaces) of joint MVPA of children and their parents [20]. As for adults, less evidence is available about the types of built environments where they actually spend their time, and what levels of PA they engage in at different locations [21,22]. Rodriguez *et al.* (2005) described adults’ PA levels indoors and outdoors within and outside the neighborhood [23]. Other studies assessed at what distance from home adults were physically active [24,25], or described the size and characteristics (fast food outlet density, number of supermarkets, and park land use) of activity spaces (*i.e.,* the subset of all locations with which individuals interact during their daily activities) in relation to PA behavior [26,27]. However, these studies used a relatively rough classification when determining the geographical locations where adults were physically active. Larson *et al.* (2014) studied a more extensive set of specific locations that were used for PA: they assessed the frequency of use of the home/backyard, neighborhood sidewalks, neighborhood parks, workplace, state parks and gyms [28], but they used self-report measures (surveys). Moreover, most studies of the effect of the environment on PA describe the distribution of PA across location types for the population as a whole, or for a limited number of subgroups. However, it is important to consider a broader set of socio-demographic factors since both PA behavior and the use of locations for PA are likely a reflection of various personal factors such as preferences and needs, access to transport options, social norms, and constraints (e.g., having children, work obligations). A proper insight into how locations are used for PA and by which population groups, requires the inclusion of a broad set of variables using multivariate analyses.

Therefore, this study aims (1) to provide insight into the locations where adults engage in MVPA; as well as (2) to assess the extent to which individual demographics influence the amount of MVPA at different locations, as well as the overall amount of MVPA.

## 2. Materials and Methods

### 2.1. Study Design, Participants and Setting

This cross-sectional study is part of PHASE (Physical Activity in public Space Environments), a research project that was conducted in the Netherlands, to investigate how PA is distributed across the home location, the neighborhood and more remote locations.

**Figure 1:**
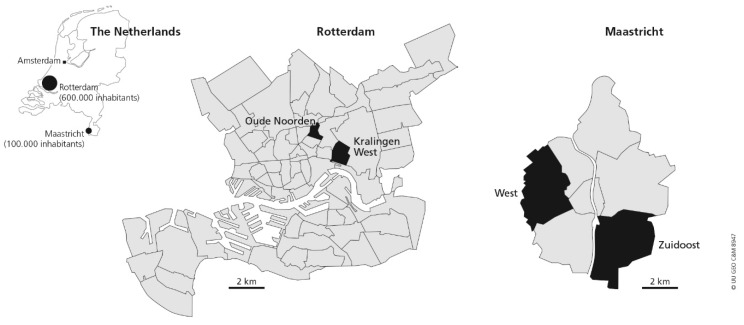
Selected neighborhoods in Rotterdam and Maastricht.

Adults aged 45–65 years were recruited in four neighborhoods in Rotterdam (623,652 inhabitants) and Maastricht (122,397 inhabitants), in the Netherlands. These neighborhoods (Kralingen-West, Oude Noorden, Zuid-Oost and West; see Figure 1) were selected based on their geographical differences, which were presence of green and parks, distance to the city center, type of buildings, and population density. Table 1 presents neighborhoods characteristics.

**Figure 2:**
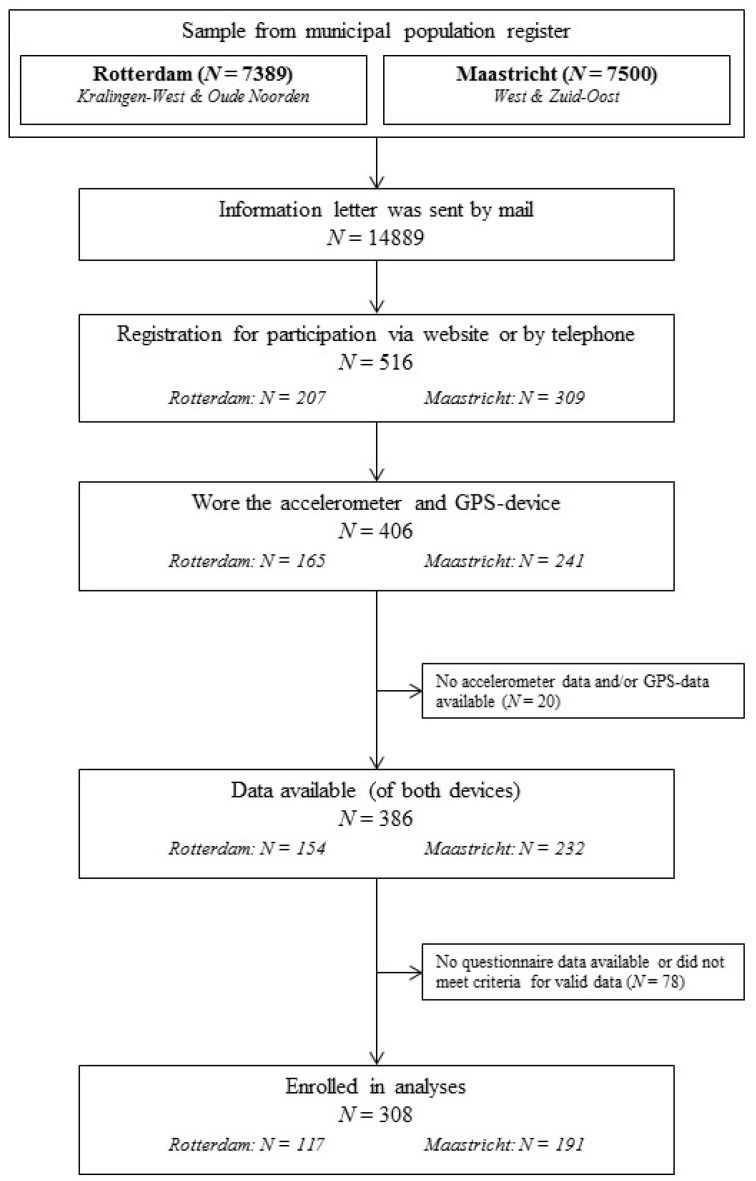
Flowchart of participant recruitment.

Addresses of inhabitants (45–65 years) were randomly selected from the municipal population registers of Rotterdam and Maastricht. Selected adults (*N* = 14,889) were contacted by an information letter in which they were asked to participate in the study. Adults who were willing to participate registered through a website or by telephone (*N* = 516). Subsequently, they were contacted by phone or e-mail to plan the distribution of an accelerometer and GPS-device. Staff members distributed the devices in community centers, and explained monitor wear and placement. Participants received an information sheet, with a summary of instructions. Data were collected between April 2014 and December 2014 and all participants signed informed consent. Participants in Rotterdam received an incentive of €10 per person, and participants in Maastricht were entered into a raffle method: 15 prizes of €100 each were raffled. Data of 308 adults could be included for analyses after applying criteria for valid data. Figure 2 illustrates the recruitment procedure.

### 2.2. Measures

**PA.** PA was objectively assessed using the Actigraph GT3X+ accelerometer (Actigraph, Pensacola, Florida, FL, USA). The accelerometer was attached to an elastic, adjustable belt. Participants were instructed to wear the device on the right hip for seven consecutive days during waking hours (except during water-based activities). Actilife v6.11.2 (Firmware 2.2.1, Actigraph, Pensacola, Florida, FL, USA) was used to download accelerometer data.

Triaxial accelerometer data were collected in 5 s epochs, and summed as counts per minute (cpm) during data processing. Vector magnitude cut-points for cpm were used to define moderate PA (3208–8564 cpm) and vigorous PA (≥8565 cpm), which were derived from a study population similar to this study’s population [30]. MVPA was calculated as the sum of moderate PA and vigorous PA.

Because of the slightly older population, non-wear time was defined as episodes of at least 90 min of consecutive zero counts [31], with allowance for up to two consecutive minutes of 1–100 cpm. Non-wear episodes ended when the cpm exceeded 100, or when three or more consecutive minutes accumulated between 1 and 100 cpm [32]. To determine the length of a valid day, the 70/80 rule was used. This rule defines a valid day as having non-missing counts for at least 80% of a measurement day [33]. A measurement day reflects the length of time in which at least 70% of all participants wore the accelerometer device [33], which was 611 min for this study. Calculating 80% of this episode of 611 min yields a valid day of 488.8 min. Only valid data of participants with at least four of these valid days were included for analyses [34].

**PA locations and trips.** Participants’ geographical locations were measured using BT-Q1000XT GPS-devices (QStarz International Co, Taipei, Taiwan). The GPS-device was attached to the same belt as the accelerometer and wearing instructions were similar to those for wearing an accelerometer. QStarz QTravel software (v1.45, Qstarz International Co., Ltd, Taipei, Taiwan) was used to download GPS-data. GPS- and accelerometer-data were date and time linked using Python software to create combinations of PA intensity and location of PA.

To gain insight into the locations where adults are actually physically active, GPS-data had to be categorized into various types of activity locations. To do so, all GPS-data were first subdivided into either stop episodes or trips. This identified a first category of activity locations, namely: trips, as being in transit can be considered a “location” where PA may take place. Trips are clusters of successive GPS data points of which (1) the average speed was 3 km/h or more; (2) the trip length was 100 m or more; and (3) the minimum duration of a trip was 1 min. “Trips” are further referred to as “transport”.

Stop episodes are clusters of successive GPS data points that met the following three criteria: (1) the maximum range of data points was 150 m; (2) the average speed of data points was under 3 km/h; and (3) the minimum duration of a stop episode was 2 min. For each stop episode, the center of gravity was calculated. The coordinates of these centers of gravity were used to map all stop episodes (stops) in ArcMap 10.2.2 (Esri, Redlands, California, CA, USA). To further categorize stops into specific types of locations, ArcMap was used to link data on land use (available from Dutch Statistics, 2010) and buildings (available from Dutch Cadaster, 2014) to these stops. For each stop, we calculated in what type of land use it was located, what types of land use (and in what proportions) occurred within a 25 meter buffer (from the center of gravity), and what functions the three nearest buildings had. Based on this information, stops were further classified into nine different categories. Table 2 shows the conditions for categorization of the stops into the activity location types “home”, “other residential area”, “residential and shopping area”, “shopping area”, “workplace”, “small green area”, “larger green area”, “sports facilities” and “other”. Since we had only access to data on land use and buildings of the Netherlands, stops with their center of gravity located outside the boundaries of the Netherlands were excluded from analyses (approximately 4% of the data). For this purpose, the centers of gravity of trips were also calculated: only trips with their center of gravity within the Netherlands were included for analyses.

**Background variables.** A questionnaire was used to collect information on home address, details on the home location (e.g., having a garden), having a car, work address, health status (SF36), and socio-demographic factors. Socio-demographic factors were age, gender, education (low, middle, high), employment (*i.e.,* yes, no), household structure (*i.e.,* having a partner, having children), neighborhood, and ethnicity (*i.e.,* autochthonous, western-, and non-western immigrants). Non-western immigrants are people who were born and/or of whom at least one parent was born in Turkey, an African country, a country in Latin-America, or in a country in Asia (except for Japan and Indonesia). Western immigrants are people who were born and/or of whom at least one parent was born in Japan, Indonesia, a European country (except for Turkey), a country in North-America, or a country in Oceania.

**Meteorological data.** Data on daily temperature (°C), sunshine (hours), and average wind speed (m/s) were obtained from Royal Dutch Meteorological Institute measurement stations in Maastricht and Rotterdam [35]. Dummy variables were created to take these variables into account in analyses. Cut-points for four equal groups were obtained using descriptive statistics in SPSS.

### 2.3. Statistical Analyses

To assess the effect of various independent variables (socio-demographic factors and neighborhood) on the interval-ratio variables total MVPA, and MVPA at different activity locations, multiple regression analyses were performed. Most of the dependent variables were not normally distributed and neither log transformations, nor taking the square root of these variables led to normal distributions. Since the normality assumption was violated, bootstrapped (resampling method) multilevel regression analyses were performed. Multilevel analyses were used to correct for clustering of days within respondents. “Income” had to be excluded from multilevel analyses to avoid multicollinearity with other variables. Statistical analyses were performed using SPSS 22.0 for windows.

## 3. Results

### 3.1. Sample Characteristics

Table 3 presents participants’ characteristics. The mean age of adults in this study sample was 56.4 (SD 6.2) years. More than half (52.9%) of participants has a healthy weight, 37.0% is overweight and 10.1% is obese. Percentages of overweight were higher in “Zuid-Oost” (45.7%) and “West” (36.0%) in Maastricht, as compared to “Oude Noorden” (25.5%) and “Kralingen-West” (33.3%) in Rotterdam. Most participants were native Dutch (84.4%). The neighborhoods of Rotterdam had the highest percentages of non-western immigrants, whereas the neighborhoods of Maastricht had the highest percentages of western immigrants. This trend is similar to percentages of immigrants in the selected neighborhoods according to Statistics Netherlands [29], but both non-western and western immigrants were underrepresented in this study population. Most participants had middle (52.9%) or higher (41.2%) education. Over 60% of the total study population was employed. In total, 1804 days were included for analyses. Participants wore the devices on average 830.7 (SD 168.1) minutes per day.

### 3.2. Average Daily PA

Total MVPA represents on average 34.0 min of the day (Table 3). Inhabitants of Oude Noorden (Rotterdam, The Netherlands) spent least time in total MVPA per day: 31.4 min, whereas inhabitants of Kralingen-West (Rotterdam, The Netherlands), Zuid-Oost (Maastricht, The Netherlands), and West (Maastricht, The Netherlands) spent 35.8, 35.3, and 33.1 min in MVPA, respectively.

### 3.3. Use of Activity Locations

Table 4 shows that all participants engaged in transport (*i.e.,* active or motorized transport) on at least one measurement day. The activity locations home, other residential area, and other were visited on at least one day by more than 90% of participants. Small green areas were visited the least (20.8%). The table also shows that most time per day is spent at home (310.6 min) and at workplaces (297.8 min). Least time per day is spent in residential- and shopping areas (18.8 min).

### 3.4. MVPA at Various Locations

Participants accumulated most minutes of MVPA at home (10.4 min) and at work (9.9 min) (Table 4). Least minutes of MVPA were accumulated in larger green areas (0.9 min) and residential and shopping areas (0.6 min). When taking total time spent at the location into account, the share of MVPA was largest in sports facilities (5.9%) and during transport (5.7%).

Table 5 shows bootstrapped multilevel regression results on the effect of neighborhood and socio-demographic determinants on total MVPA and on MVPA at various locations.

**Total MVPA.** Negative correlates of total MVPA were being a western immigrant, fair, poor, and very poor health status, overweight and obesity, employment, middle and higher education, having children ≤4 years, having children aged 11–17 years, having a garden at home, and a wind speed of 2.5–3.3 m/s. Positive correlates were having a dog, having a garden elsewhere (e.g., allotment), weekend days, living in Zuid-Oost or West, and higher temperatures (≥13.2 °C).

**Home.** Being a western immigrant, having a good or fair health status, being overweight or obese, being employed and having children aged ≤4 years, were negatively associated with MVPA at home. Aged 56–60 years, aged >60 years, being female, having an employed partner, having a dog, weekend days, living in Kralingen-West, Zuid-Oost or West, and higher temperatures (≥7.6 °C), were all factors that were positively associated with MVPA at home.

**Other residential area.** MVPA in other residential area was negatively associated with age >60 years, western and non-western ethnicity, good health status, fair health status, poor health status, very poor health status, employment, having a partner, and having a garden at home, whereas it was positively associated with being female, having a dog, and living in Kralingen-West or West.

**Residential- and shopping area.** Having children aged ≤4 years and living in the neighborhood West were negative correlates of MVPA at this location type.

**Shopping area.** Negative correlates of MVPA in shopping area were being a western immigrant, good health status, fair health status, very poor health status, obesity, middle and higher education, having children aged ≤4 years, having a dog, and living in Kralingen-West, Zuid-Oost or West, whereas weekend was a positive correlate.

**Workplace.** Being non-western immigrant, having a higher education, and living in Kralingen-West, Zuid-Oost or West, negatively affected MVPA at the workplace. Positive effects were found for having ≥2 cars.

**Small green area.** Aged >60 years, having children aged ≤4 years, and car ownership were negatively associated with MVPA in small green area, whereas having a garden at home or elsewhere was positively associated with MVPA in small green area.

**Larger green area.** Negative correlates of MVPA in larger green area were western- and non-western ethnicity, having a dog, and living in the neighborhood Zuid-Oost or West. Positive correlates were car ownership, and temperatures between 7.6 and 16.6 °C.

**Sports facilities.** Having children aged 4–11 years negatively influenced MVPA at sports facilities. Having children aged ≤4 years and living in the neighborhoods Kralingen-West, Zuid-Oost, or West, positively influenced MVPA at sports facilities.

**Transport.** Negative correlates of MVPA during transport were western and non-western ethnicity, fair, poor, and very poor health status, overweight and obesity, being employed, being female, having children aged 4–11 years, having children aged 11–17 years, having ≥2 cars, having a garden at home, and a wind speed of 2.5–3.3 m/s. Positive correlates of MVPA during transport were having a dog, weekend days, living in Zuid-Oost or West, and 0.3–2.8 h of sunshine.

**Other.** Very poor health status, overweight and obesity, higher education, having a partner, having an employed partner, having children aged ≤4 years, and living in Kralingen-West, Zuid-Oost or West were negative correlates of MVPA at other locations. Positive correlates were fair health status, poor health status, having children aged 4–11 years, having ≥2 cars, and having a garden at home.

## 4. Discussion 

### 4.1. Main Findings

This study addressed the need for more detailed and comprehensive insight in objectively measured PA behavior at various locations [21,28]. By assessing PA behavior of adults in a much wider variety of locations than existing studies, and by investigating the effect of a variety of socio-demographic characteristics on PA levels at specific locations, this paper expands existing literature.

Consistent with other studies [36,37,38], the current study found that adults spent on average 34.0 min (approximately 4% of wear time) per day in MVPA. In congruence with the literature, we found that ethnicity, poorer health status, overweight/obesity, and having children were negative correlates of MVPA [39], and that weekend days and having a dog were positive correlates of MVPA [40,41]. The finding that adults with a middle or higher education had lower levels of MVPA was, however, in contrast with literature since most studies found that lower educated adults have lower levels of MVPA [39]. Besides, it is remarkable that having a garden at home had a negative effect on total MVPA, whereas having a garden elsewhere positively affected total MVPA. To improve understanding of MVPA behavior, we refined MVPA behavior into MVPA behavior at various locations and assessed whether correlations between various factors and MVPA were also found for those locations. MVPA behavior was distributed across many different locations, but most time in MVPA was spent at home and work and least time in MVPA was spent in larger green areas and residential- and shopping areas.

This study found that the home location was an important contributor to PA, which is consistent with findings of the Eurobarometer on Sports and PA, that shows that the home location is the second most common location for PA and sports (after parks and outdoors) [42]. The home is also a place where adults spent most of their total wear time per day, and it may thus be that when total time spent at a location increases, time spent in MVPA at that location also increases. Obviously, this does not mean that staying at home is the solution for increasing MVPA levels, since other locations (e.g., sports facilities and green areas) can be important facilitators for PA as well [43,44]. MVPA behavior at home was found to be higher for adults of older age. Additional analyses may explain this as they show that adults of the two oldest age groups spent significantly more time at home per day, than adults of the two youngest age groups, and therefore accumulated more minutes of MVPA there. Increased levels of MVPA at home were also found for females and adults with an employed partner. This may be due to the influence of role expectations [45]: women may accumulate more minutes of MVPA at home by doing more household activities then men, and an individual whose partner is employed may have to do more household activities because the partner has less time for those activities.

The work location is after the home location, the place where adults spent most MVPA minutes. Adults with a higher education spent less time in MVPA at work than adults with a lower education. An explanation for this may be that adults with a higher education more often have jobs that require them to be seated behind a desk and computer, whereas adults with a lower education might more often have jobs that require them to be physically active. This explanation is supported by existing literature which states that for some occupations (e.g., service workers), the workplace is an important source for total PA, whereas other occupancies or sectors require much sedentary work with only limited PA throughout the day (e.g., computerization) [46,47].

Shopping areas are places where adults spent more MVPA time on the weekends. On weekend days, adults may experience less time constraints and may thus have more opportunities to visit places other than their home or workplace. Besides, shops in the Netherlands (except for grocery stores) close at approximately the same time a working day ends, which also hinders adults in visiting those locations on weekdays.

Green spaces can be important facilitators of PA behavior [44], but varying types of green spaces can contribute to MVPA of diverse subpopulations in different ways. For example, this study found that MVPA levels in small green area were lower for car owners than for adults who do not own a car. On the other hand, levels of MVPA in larger green area were higher for car owners than for adults who do not own a car. This may be explained by previous studies that showed that car owners tend to undertake more PA outside their residential neighborhood [25]. As small green areas (*i.e.,* parks and allotments) are likely to be present within one’s residential neighborhood, and larger green area (*i.e.,* forests, recreational areas, moorland) are often located further from the home, it is plausible that car owners use their cars to visit larger green areas and be physically active there, whereas adults without a car visit small green areas within their neighborhood, which they can reach by foot or bike. Not having a car may thus be a constraint for adults to engage in MVPA in larger green areas, but also in other places. Interestingly, living in the neighborhoods Zuid-Oost and West (Maastricht) was negatively correlated to MVPA in larger green areas, whereas these neighborhoods have the highest amounts of larger green areas as compared to the neighborhoods of Rotterdam. As we found that car owners spent more time in larger green areas, proximity of and travel distance to larger green spaces may thus become of less importance.

MVPA at sports facilities was lowest for adults living in Oude Noorden (Rotterdam). They also spent least time in sports facilities, compared to participants of the other three neighborhoods. This may be influenced by the presence of sports facilities in one’s neighborhood, as the amount of sports facilities within the neighborhood Oude Noorden are lower than in Kralingen-West, Zuid-oost and West. Visual analyses of data on MVPA behavior at sports facilities indicated that adults may have taken off the devices during exercise, as we identified gaps in time. Since data before and after such time gaps indicated that participants were at a sports facility, it seems plausible that during the gap, participants were also at that facility. Results may thus underestimate MVPA levels at sports facilities.

Other residential areas were positively correlated to MVPA behavior of dog owners. This finding is not unexpected, as dog owners are likely to walk their dog in residential areas such as the residential neighborhood or adjacent neighborhoods. Adults of older age and adults with a poorer health status spent less time in MVPA in other residential areas. An explanation for this may be that these adults experience physical limitations (e.g., difficulties with walking) that hinder them to be physically active in these areas. In addition, employed adults spent less time in MVPA in other residential areas, which may be explained by the obligation to spend a certain amount of time at the workplace.

We found that transport-related MVPA differs between native Dutch adults, and western and non-western immigrants. This is supported by figures on cycling in the Netherlands, which show that native Dutch adults cycle more often than individuals with other ethnic origins [48]. Additional analyses revealed that compared to autochthonous adults, significantly less non-western immigrants have a bicycle. Here, cultural differences seem to influence MVPA behavior. Physical limitations seem to be of influence as well, as we found that adults who reported lower levels of health status had lower levels of transport-related MVPA. It may be that adults who reported a very good health status had less difficulties with walking and cycling than adults with a lower health status, for example, because they felt better, or had less health complaints. On the other hand, it may also be that their engagement in MVPA during transport contributed to a better health status.

This study found that some neighborhood, weather, and socio-demographic were correlates of both total MVPA and MVPA at various locations, whereas other factors were found to be only correlates of MVPA behavior at specific locations. The first option not only provides information on the correlates of MVPA behavior, but in addition contributes to the explanation of these findings. The other option, that factors were only associated with MVPA at specific locations, is likely due to substitution effects (*i.e.,* an increase in MVPA levels at one location may lead to a decrease of MVPA at another location). For example, adults of older age spent more time in MVPA at home, whereas they spent less time in MVPA in other residential areas and small green areas. Another example is that women have higher levels of MVPA at home and in other residential areas, whereas their levels of transport-related MVPA were lower. Preferences of such subpopulations may be one reason for this substitution effect to occur, since it is likely that preferences and needs vary between adults with different constraints and different socio-demographic characteristics. Moreover, constraints such as work obligations, taking care of children, or not having a car, may also be a reason for this substitution effect to occur. It may be due to these substitution effects that no effects of those factors on total MVPA were found.

### 4.2. Limitations

This study has some limitations, such as a relatively low response rate, data loss, underestimation of specific behaviors, and unequivocal categorization of activity locations. Although response rates were low (3.5% of the 14,889 randomly recruited individuals agreed to participate, and 78.7% of these individuals actually wore the devices), our final study sample is comparable to other studies [33,49]. Data loss was due to the inability to match all GPS and accelerometer data points, insufficient wear time, and the interference of urban canyons (surrounding high buildings), trees, or building materials (e.g., in a tunnel or at home) with satellite communication. Besides, wearing the accelerometer on the hip may have led to an underestimation of PA, since upper-body movements or non-ambulatory movements (e.g., cycling) are less accurately recorded [50]. The locations were classified based on land use and building data, but classification is not unequivocal. For example, it is likely that walking the dog in larger green areas was classified as transport, and not as an activity that took place in larger green areas. Although this is not misclassification, since it is both correct, future research may consider further classifying transport into categories (e.g., transport in larger green areas). Besides, a diary kept by participants may improve accuracy of the determination of activity locations. Another limitation may be that determinants, which were not controlled for in this study, also determine the effect of neighborhood on PA. For example, social norms were significant predictors of PA in previous studies [51,52]: individuals who often saw other people exercising or walking in their neighborhood had higher levels of leisure-time MVPA and walking than individuals who did not often see other people exercising and walking in their neighborhood [52].

## 5. Conclusions

The general conclusions drawn from this study are that (a) adults’ MVPA is distributed across a variety of location types, including locations that have not received much attention in urban policy, such as the home and work location, and (b) the relative importance of location types differs with factors such as car ownership, work status, health status and household structure. These insights can be used to target specific population groups by making location specific environmental changes. This may increase these groups’ levels of PA and, therefore, reduce inequalities across the total population in terms of PA and therefore health. Future studies may investigate what specific (environmental) characteristics (e.g., green, residential density) of these different locations are facilitators of, or barriers to, PA. In this line of research, insight into the role of locations that are important for PA, their accessibility, but also their affordances or barriers in PA behavior, may benefit from combining objectively collected data (*i.e.,* by accelerometer and GPS-device) with subjective data of individual perceptions of, or experiences with, PA at specific locations. Moreover, the relatively small amounts of MVPA at different locations make it plausible to assume that other intensity levels of PA, such as light PA (LPA), also play a role at those locations. For adults aged 45–65 years, LPA may be more feasible and easier to implement in daily life. Since LPA has recently been positively associated with health [53], it would be of great interest for future research to investigate both LPA and MVPA in relation to different location types.

## Figures and Tables

**Table 1:** Neighborhood characteristics.

Characteristics	Rotterdam		Maastricht	
	*Oude Noorden*	*Kralingen-West*	*Zuid-Oost*	*West*
Surface area *(hectometer)*	107	102	1089	884
Land	101	102	970	884
Water	6	0	119	0
Inhabitants *(N)*	16,815	15,115	21,760	37,865
Population density *(N inhabitants per km*^2^*)*	16,658	14,778	2244	4285
Housing density *(N addresses per km*^2^*)*	≥2500	≥2500	1500–2500	1500–2500
Land use (%)				
Residential area	69.3	85.7	26.1	51.6
Parks, public garden	0.0	3.1	3.5	5.9
Agricultural area, recreational area, forest	0.0	0.0	34.2	19.9
Sports facilities	0.0	3.1	2.2	12.2
Roads, streets	7.8	0.2	6.6	3.0

Source: Statistics Netherlands [29].

**Table 2:** Categorization of stops into various activity locations.

Activity Locations (AL)	AND/OR	Classification Conditions
Home	OR	AL within 25 meter buffer from home address, obtained through questionnaire. AL within 25 meter buffer from home address, obtained by using GPS-data: if the first and last coordinates of multiple days were identical, these coordinates were considered the home location—only when the home address missed or was incomplete
Other residential area		BF_25_ = residence
	AND	BF_25_ ≠ shops or foodservice industry
	AND	LU_25_ = residences > 70%
Residential & shopping area		BF_25_ = shops or foodservice industry
	AND	BF_25_ = residence
	AND	BF_25_ ≠ other functions
Shopping area		BF_25_ = shops or foodservice industry
	AND	BF_25_ ≠ other functions
	OR	BF_25_ = shops or foodservice industry
	AND	LU_0_ = shops or foodservice industry
	AND	LU_25_ = shops or foodservice industry > 70%
Workplace	OR	AL within 50 meter buffer from work address obtained through questionnaire. AL located within 25 meter from health care institutions, offices, educational institutions, lodging, industry or shops according to building data, and if participants spent at least 240 min at that location.
Small green area		LU_0_ = parks and public garden or allotment garden
Larger green area		LU_0_ = recreational area, agricultural area, forest, or natural terrain
Sports facilities	OR	LU_0_ = sport terrain BF_10_ = sport facility *Sports facilities in the Netherlands require membership or subscription, this comes with certain costs that differ per type of sport.*
Other		If not classified as any other category

Note: BF_25_ = Building function within 25 meter buffer from stop. BF_10_ = Building function within 10 meter buffer from stop. BF_50_ = Building function within 50 meter buffer from stop. LU_0_ = Type of land use in which the stop is located. LU_25_ = Type(s) of land use (%) within a 25 meter buffer around the stop.

**Table 3:** Population characteristics.

Characteristics	Total Study Sample (*N* = 308)	Oude Noorden (*N* = 51) (Rotterdam)	Kralingen-West (*N* = 66) (Rotterdam)	Zuid-Oost (*N* = 105) (Maastricht)	West (*N* = 86) (Maastricht)
**Age** in years *Mean (SD)*	56.4 (6.2)	55.1 (5.9)	55.5 (5.8)	56.7 (6.3)	57.6 (6.4)
**Female** (%)	54.9	62.7	50.0	58.1	50.0
**BMI** (%)					
Healthy weight	52.9	60.8	62.1	43.8	52.3
Overweight	37.0	25.5	33.3	45.7	36.0
Obese	10.1	13.7	4.5	10.5	11.6
**Ethnicity** (%)					
Autochthonous	84.4	82.4	80.3	86.7	86.0
Western immigrants	6.8	5.9	4.5	7.6	8.1
Non-Western immigrants	7.5	11.8	13.6	3.8	4.7
Missing	1.3	0	1.5	1.0	1.2
**Education** (%)					
Lower	4.2	7.4	4.5	3.8	2.3
Middle	52.9	54.9	27.3	59.0	64.0
Higher	41.2	35.3	65.2	37.1	31.4
Missing	1.6	2.0	3.0	0	2.3
**Employment** (%)					
Employed	61.7	68.6	65.2	60.0	57.0
Not employed	37.3	31.4	33.3	39.0	41.9
Missing	1.0	0	1.5	1.0	1.2
**Days included in analyses** (*N*)	1804	282	380	626	516
**Wear time** in minutes per day *Mean (SD)*	830.7 (168.1)	823.9 (159.4)	832.3 (159.4)	833.5 (169.0)	829.9 (178.0)
**Levels of MVPA** in minutes per day *Median (IQR)*	34.0 (38.8)	31.4 (39.4)	35.8 (38.7)	35.3 (40.8)	33.1 (36.9)

**Table 4:** Total time spent, and time spent in MVPA at various locations by neighborhood.

Location Types	Total Study Sample (*N* = 308)		Oude Noorden (*N* = 51) (Rotterdam)		Kralingen-West (*N* = 66) (Rotterdam)		Zuid-Oost (*N* = 105) (Maastricht)		West (*N* = 86) (Maastricht)	
**Home**										
Adults who visited the location ^a^ (%)	94.5		96.1		97.0		92.4		94.2	
Time spent at the location ^b^ (min/day)	310.6	(352.0)	246.4	(303.6)	317.8	(371.0)	348.0	(355.8)	295.9	(316.0)
Time spent in MVPA at the location (min/day)	10.4	(16.8)	9.2	(17.0)	11.5	(21.0)	11.8	(17.4)	8.8	(14.6)
MVPA-ratio of total time spent at location ^c^ (%)	3.8	(4.1)	4.0	(5.3)	4.2	(5.2)	3.8	(3.6)	3.7	(4.2)
**Other residential area**										
Adults who visited the location ^a^ (%)	94.2		96.1		90.9		94.3		95.3	
Time spent at the location ^b^ (min/day)	116.9	(222.5)	131.5	(171.1)	141.3	(218.9)	105.3	(232.3)	108.4	(254.7)
Time spent in MVPA at the location (min/day)	5.0	(14.2)	6.1	(12.0)	5.8	(15.8)	4.2	(13.5)	4.7	(15.9)
MVPA-ratio of total time spent at location ^c^ (%)	4.5	(6.3)	4.5	(7.3)	4.6	(6.1)	4.3	(5.9)	4.5	(6.6)
**Residential and shopping area**										
Adults who visited the location ^a^ (%)	40.6		41.2		33.3		49.5		34.9	
Time spent at the location ^b^ (min/day)	18.8	(56.6)	18.3	(122.0)	55.4	(150.1)	20.9	(52.6)	9.8	(15.9)
Time spent in MVPA at the location (min/day)	0.6	(2.8)	0.4	(5.9)	2.3	(5.8)	0.8	(2.2)	0.3	(0.9)
MVPA-ratio of total time spent at location ^c^ (%)	2.9	(6.6)	3.0	(7.0)	2.2	(5.0)	4.2	(6.7)	2.4	(5.6)
**Shopping area**										
Adults who visited the location ^a^ (%)	76.3		64.7		80.3		80.0		75.6	
Time spent at the location ^b^ (min/day)	21.6	(52.0)	53.1	(149.7)	12.4	(33.4)	20.7	(42.9)	26.5	(52.6)
Time spent in MVPA at the location (min/day)	1.0	(4.1)	2.0	(9.2)	0.3	(2.6)	1.0	(3.8)	1.4	(4.5)
MVPA-ratio of total time spent at location ^c^ (%)	5.0	(10.6)	4.0	(9.1)	2.8	(8.0)	6.1	(13.0)	5.3	(12.1)
**Small green area**										
Adults who visited the location ^a^ (%)	20.8		27.5		42.4		9.5		14.0	
Time spent at the location ^b^ (min/day)	24.0	(160.5)	97.0	(241.9)	22.9	(115.3)	26.3	(95.0)	13.1	(124.5)
Time spent in MVPA at the location (min/day)	1.0	(10.1)	2.5	(18.0)	1.0	(12.6)	0.3	(5.9)	0.6	(9.6)
MVPA-ratio of total time spent at location ^c^ (%)	4.5	(10.9)	3.5	(10.2)	5.3	(14.2)	3.0	(13.8)	4.2	(5.4)
**Larger green area**										
Adults who visited the location ^a^ (%)	43.2		35.3		34.8		57.1		37.2	
Time spent at the location ^b^ (min/day)	35.6	(106.9)	76.1	(206.0)	139.3	(338.1)	28.8	(74.1)	21.8	(67.8)
Time spent in MVPA at the location (min/day)	0.9	(6.6)	6.2	(24.6)	7.0	(24.1)	0.7	(2.4)	0.3	(1.1)
MVPA-ratio of total time spent at location ^c^ (%)	3.6	(8.8)	7.2	(18.7)	5.3	(8.8)	3.8	(10.5)	2.1	(4.9)
**Sports facilities**										
Adults who visited the location ^a^ (%)	36.0		23.5		36.4		35.2		44.2	
Time spent at the location ^b^ (min/day)	73.6	(128.9)	31.4	(78.4)	89.9	(216.5)	69.3	(115.5)	75.6	(105.3)
Time spent in MVPA at the location (min/day)	4.2	(19.6)	2.8	(7.6)	9.3	(22.1)	2.5	(16.9)	6.0	(29.6)
MVPA-ratio of total time spent at location ^c^ (%)	5.9	(21.6)	5.0	(12.5)	6.8	(17.7)	4.0	(20.2)	9.5	(26.0)
**Workplaces**										
Adults who visited the location ^a^ (%)	46.1		51.0		50.0		43.8		43.0	
Time spent at the location ^b^ (min/day)	297.8	(349.0)	333.0	(329.7)	260.7	(365.7)	285.3	(378.8)	319.1	(311.9)
Time spent in MVPA at the location (min/day)	9.9	(19.6)	13.5	(26.8)	9.8	(20.6)	8.3	(11.1)	14.0	(17.9)
MVPA-ratio of total time spent at location ^c^ (%)	4.2	(5.2)	5.2	(7.4)	3.6	(4.9)	4.1	(7.4)	4.3	(3.8)
**Other**										
Adults who visited the location ^a^ (%)	96.8		100.0		100.0		97.1		91.9	
Time spent at the location ^b^ (min/day)	46.9	(129.9)	77.7	(152.5)	45.3	(126.7)	39.0	(120.8)	42.3	(117.3)
Time spent in MVPA at the location (min/day)	1.8	(7.1)	3.6	(10.8)	2.1	(8.0)	1.4	(5.7)	1.4	(5.6)
MVPA-ratio of total time spent at location ^c^ (%)	4.3	(8.5)	5.3	(8.9)	4.2	(8.2)	4.2	(8.1)	3.8	(9.3)
**Transport**										
Adults who visited the location ^a^ (%)	100.0		100.0		100.0		100.0		100.0	
Time spent at the location ^b^ (min/day)	78.5	(89.9)	72.4	(86.6)	86.0	(98.4)	79.0	(89.1)	76.4	(85.7)
Time spent in MVPA at the location (min/day)	4.6	(11.4)	3.6	(8.4)	4.1	(9.2)	5.3	(15.1)	5.1	(13.8)
MVPA-ratio of total time spent at location ^c^ (%)	5.7	(10.9)	4.5	(6.6)	5.0	(7.4)	6.6	(14.7)	6.8	(14.4)

Note: Time spent (in MVPA) and MVPA-ratio is represented as medians and interquartile ranges: median (IQR). ^a^ On at least one day during the time of measurement. ^b^ Calculated over the days that participants were actually at the specific location. ^c^ MVPA-ratio was calculated as time spent in MVPA at the location (minutes) divided by total time spent at that location (minutes), and then multiplied by 100%.

**Table 5:** Associations between individual determinants, type of day, weather, neighborhood, and daily MVPA.

Variables	Total ^b^	Home ^b^	Other Residential Area ^b^	Residential & Shopping Area ^f^	Shopping Area ^b^	Work-Place ^b^	Small Green Area ^f^	Larger Green Area ^f^	Sports Facilities ^f^	Transport ^b^	Other ^b^
Intercept	50.23	8.73	18.31	3.53	17.48	21.17	14.83	4.63	5.54	11.64	14.37
**Socio-demographics**											
Age (Ref.: 45–50 years)											
51–55 years		+0.16	−0.06				−0.77				
56–60 years		+2.13 *	+1.20				−4.88				
>60 years		+2.08 ^*^	−2.94 **				−7.61 **				
Ethnicity (Ref.: autochthonous)											
Western	−10.69 **	−3.43 **	−2.68 **		−3.07 **	−3.26		−3.82 **		−4.64 **	
Non-Western	+1.43	−0.31	−2.12 *		−0.12	−3.32 **		−4.82 *		−1.99 **	
**Health-related determinants**											
Health status (Ref.: Very good)											
Good	−2.40	−3.90 **	−2.32 **		−3.65 **					+0.52	+0.59
Fair	−5.19 **	−2.21 **	−3.74 **		−2.62 **					−3.21 **	+6.00 **
Poor	−9.70 **	−0.17	−6.52 **		−2.40					−3.66 **	+6.82 **
Very poor	−27.83 **	−2.71	−12.08 **		−6.20 **					−5.40 **	−3.03 **
BMI (Ref.: healthy weight)											
Overweight	−10.02 **	−3.98 **			−0.56					−4.51 **	−2.28 **
Obesity	-11.90 **	−7.45 **			−2.68 **					−2.89 **	−5.43 **
**Work and education**											
Employed	−6.67 **	−3.98 **	−6.11 **							−2.29 **	
Education (Ref.: lower education)											
Middle	−6.48 **				−7.69 **	+0.80					−3.06
Higher	−5.72 *				−10.29 **	−6.17 **					−4.66 **
**Household structure**											
Female		+2.48**	+1.68 **			NA				−3.30 **	
Having a partner			−1.87 **			NA					−2.09 **
Partner is employed		+3.53**				NA					−1.84 **
Having a child aged ≤ 4	−16.41 **	−7.18**		−2.33**	−3.17 **	NA	−4.42 *		+5.62 **		−8.03 **
Having a child aged 4–11						NA			−10.74 **	−2.87 **	+3.56 *
Having a child aged 11–17	−2.29 *					NA				−2.73 **	
Having a dog	+16.11 **	+7.19**	+5.32 **		−3.23 **	NA		-4.21 **		+4.28 **	
**Car ownership, garden**											
Car ownership (Ref.: no car)											
1 car						+0.43	−11.61 **	+7.17 **		+1.32	+0.92
≥ 2 cars						+5.69 **	−16.11 **	+8.33 **		−2.59 **	+5.01 **
Having a garden (Ref.: no garden)						NA					
Garden at home	−3.83 **		−3.60 **				+5.70 **			−1.55 **	+1.82 **
Garden elsewhere	+9.53 **		−1.43				+21.21 **			+2.32	+2.30
**Day of the week**											
Weekend day (Ref.: weekday)	+8.76 **	+4.43 **			+2.17 *	NA				+6.41 **	
**Neighborhood**											
Neighborhood (Ref.: Oude Noorden)											
Kralingen-West	+1.70	+7.17 **	+2.19 *	+5.44	−4.27 **	-4.15 *		−1.40	+8.95 **	+0.20	−3.73 **
Zuid-Oost	+8.68 **	+7.17 **	+0.29	−0.58	−2.74 **	-8.63 **		−8.80 *	+6.91 **	+7.47 **	−6.14 **
West	+5.55 *	+3.65 *	+2.44 **	−2.08 *	−3.20 **	-7.06 **		−11.59 **	+10.04 **	+5.32 **	−6.19 **
**Weather**											
Max. temperature (°C) (Ref.: ≤ 7.6°C) ^a^											
7.6 < °C ≤ 13.2	+0.77	+2.61 **						+3.11 *			
13.2 < °C ≤ 16.6	+5.56 *	+3.66 **						+6.87 **			
°C > 16.6	+6.17 *	+3.66 *						+6.72			
Sunshine (hours) (Ref.: h ≤ 0.3) ^a^											
0.3 < h ≤ 2.8										+2.03 *	
2.8 < h ≤ 6.9										+1.29	
h > 6.9										+0.46	
Wind speed (m/s) (Ref.: ≤ 2.5) ^a^											
2.5 < m/s ≤ 3.3	−5.14 **									−3.66 **	
3.3 < m/s ≤ 4.3	+2.34									−0.14	
m/s > 4.3	+2.30									+0.17	

Note: * *p*-value < 0.1, ** *p*-value < 0.05. NA = not assessed. ^a^ Dummy variables were created based on cut off values for 4 equal categories, as obtained in SPSS. ^b^ Backward selection procedure. ^f^ Forward selection procedure: backward procedure could not be performed since this blew up the model.
